# Clinical-demographic profile, aggravating factors, comorbidities, and quality of life in patients with Rosacea: a Brazilian multicenter study (GBPER: Brazilian Research and Studies Group on Rosacea)^☆^

**DOI:** 10.1016/j.abd.2025.501160

**Published:** 2025-07-11

**Authors:** Renan Rangel Bonamigo, Paula Barea, Juliano Peruzzo, Juliana Boza, Hélio Amante Miot, Edileia Bagatin, Luiz Maurício Costa Almeida, Giovanni Indelicato Milano, Carla Wanderley Gayoso de Lima, Linácia Freitas Vidal, Fabíola Rosa Picosse, Bruna Manuella de Figueiredo Afonso, Clivia Maria Moraes de Oliveira

**Affiliations:** a Service of Dermatology, Hospital de Clínicas de Porto Alegre, Faculty of Medicine, Universidade Federal do Rio Grande do Sul, Porto Alegre, RS, Brazil; b Service of Dermatology, Santa Casa de Porto Alegre, Porto Alegre, RS, Brazil; c Universidade Federal do Rio Grande do Sul, Porto Alegre, RS, Brazil; d Department of Dermatology, Faculdade de Medicina, Universidade Estadual Paulista, Botucatu, SP, Brazil; e Department of Dermatology, Escola Paulista de Medicina, Universidade Federal de São Paulo, São Paulo, SP, Brazil; f Dermatology Clinic, Santa Casa de Misericórdia Belo Horizonte, Belo Horizonte, MG, Brazil; g Department of Infectious, Parasitic and Inflammatory Diseases, Universidade Federal da Paraíba, João Pessoa, PB, Brazil; h Department of Dermatology, Universidade Federal do Pará, Belém, PA Brazil

**Keywords:** Acneiform eruptions, Classification, Epidemiology, Facial dermatoses, Rosacea

## Abstract

**Background:**

Rosacea is the most prevalent chronic vascular-inflammatory dermatosis of the face. Its pathogenesis includes genetic and environmental factors, neurovascular alterations, and innate immunity. Many triggering and aggravating factors, as well as systemic comorbidities, have been associated with the disease, but there are few studies on its epidemiology in Brazil.

**Objectives:**

To describe the profile of patients with rosacea treated at referral centers, as well as to investigate the presence of comorbidities, dietary aspects, worsening factors, and quality of life.

**Methods:**

Cross-sectional and multicenter Brazilian study. Clinical and demographic data, disease severity, triggering and/or aggravating factors, diet, comorbidities, and impact on quality of life were evaluated.

**Results:**

258 patients were included, predominantly women, between 35 and 65 years old and phototypes III, IV and II. The clinical picture ranged from mild to moderate in 89% of cases and quality of life was reasonable to slightly affected in 58% of cases. Aggravating factors for rosacea were reported by 96% of patients, with climate exposure, alcoholic beverages, and emotional changes being the most frequent. Among the foods mentioned as aggravating factors (28%), pepper, other condiments and hot beverages were the most frequently reported. Comorbidities were reported by 89% of the participants, with emphasis on endocrine (48%), psychiatric (35%), cardiovascular (31%) and gastrointestinal (28%) diseases.

**Study limitations:**

Uncontrolled study, including patients undergoing dermatological treatment.

**Conclusions:**

This study establishes that the profile of Brazilian patients with rosacea corroborates that described in the literature, with the presence of the disease in higher phototypes being relevant. Pepper and other condiments and hot beverages were important aggravating factors, and the presence of various comorbidities was reported by most of the patients.

## Introduction

Rosacea is a chronic inflammatory skin disease that mainly affects the central regions of the face (malar region, nose, eyes, chin and forehead). It mainly affects women with fair skin and aged between 20 and 50 years, although children can be affected.^1^ The prevalence of the disease varies between the studied countries and according to ethnic composition, ranging from 1% to over 20% of adults.^2^, ^3^, ^4^ In Brazil, there are no population-based studies; a recent study carried out in 13 dermatology outpatient clinics estimated a prevalence of 13% among adult dermatological patients.^5^ Although being more common in women, men more frequently develop more severe forms of the disease, especially phymas.^6^

Clinically, it is characterized by persistent facial erythema with or without edema, papules, pustules, recurrent episodes of transient erythema (flushing), telangiectasias and phymas.^1^, ^7^ It is believed that half of the patients have ocular involvement; however, ophthalmological complaints are often underestimated.^8^

The diagnosis of rosacea is clinical and does not require laboratory tests. Anatomopathological examination may occasionally be useful to rule out other diseases. Several diagnostic systems have been proposed; however, the most widely used is the one proposed by the Global Rosacea Consensus (ROSCO) panel, with criteria based on the disease phenotype.^9^, ^10^, ^11^, ^12^

Rosacea negatively affects quality of life due to its chronicity, irritative symptoms, evolution in flare-ups, and because it affects the face. It can cause embarrassment, anxiety, low self-esteem, harm to professional relationships, and even difficulty finding a job.^13^, ^14^, ^15^

Its pathogenesis is complex and not yet fully understood, including genetic predisposition and other intrinsic and extrinsic factors related to the disease phenotype.^16^ The participation of the cutaneous (e.g., increased colonization by the *Demodex* sp. mite) and gastrointestinal microbiomes has gained recent importance.^17^ The association of diet with rosacea is controversial; some foods may cause exacerbation or exert possible protection through regulation of the intestinal microbiome.^18^, ^19^

Neurovascular dysregulation may be related to the occurrence of flushing. The mediators suggested as markers of this mechanism include vascular endothelial growth factor (VEGF) and CD 31.^20^ Ultraviolet radiation (UVR) can induce an increase in cutaneous VEGF, exacerbating rosacea.^21^

The participation of innate immunity is evidenced by the greater expression of the Antimicrobial Peptide (AMP), of the cathelicidin family, of 37-amino-acid peptide (LL-37), the enzyme that activates it - a serine protease of the kallikrein family - produced by keratinocytes, and of the Toll-Like-2 receptor (TRL-2) in skin lesions.^10^, ^20^, ^21^, ^22^, ^23^, ^24^, ^25^, ^26^

Moreover, the skin of the face in patients with rosacea is very sensitive, with alterations in the skin barrier that favor inflammation.^27^, ^28^

Several factors are reported as triggers or exacerbators of rosacea, such as stress, hot or cold climates, wind, solar radiation, ingestion of hot beverages, alcoholic beverages, tea, coffee, spicy foods and smoking.^29^

Rosacea has been associated with several systemic comorbidities: neurological, psychiatric, gastrointestinal and cardiovascular ones.^29^, ^30^ Its association with metabolic syndrome may result from increased cathelicidins, pro-inflammatory cytokines and systemic oxidative stress.^31^

Despite its relevance, there is no comprehensive Brazilian data on different aspects of the profile of patients with rosacea. The scarce studies include few variables; there are also no publications investigating associated comorbidities, dietary habits, and the disease impact on quality of life.^5^, ^32^

This study aimed to describe the clinical and demographic profile of patients with rosacea, as well as to investigate comorbidities, dietary habits, perception of worsening factors, and the disease impact on quality of life, in samples from different regions of Brazil.

## Methods

A cross-sectional, descriptive, multicenter study was conducted in the Dermatology Services (all accredited by the Brazilian Society of Dermatology), by the Brazilian Research and Studies Group on Rosacea, of the following institutions in Brazil: Universidade Federal do Rio Grande do Sul/Hospital de Clínicas de Porto Alegre; Universidade Federal de São Paulo; Universidade Estadual Paulista Faculty of Medicine; Santa Casa de Belo Horizonte, Universidade Federal da Paraíba and Universidade Federal do Pará, between March 2022 and September 2023. The research was approved by the Ethics and Research Committees of each center.

The inclusion criteria were: adult patients diagnosed with rosacea by certified dermatologists. The exclusion criterion used was the presence of other facial dermatoses (other than rosacea) that prevented a correct clinical evaluation at the time of the consultation.

The calculations to define the sample size were performed using the WINPEPI 11.65® program. To estimate the frequency of comorbidities of 80% in patients with rosacea, with a margin of error of 5 percentage points (acceptable difference = 0.05) and a confidence level of 95%, 246 study participants were required.

After signing the Informed Consent Form (ICF), the participants answered data collection instruments: a clinical data questionnaire, including Body Mass Index (BMI), demographic data and comorbidities, a questionnaire on factors that aggravate rosacea, a dietary questionnaire focusing on factors that trigger rosacea, and a quality of life questionnaire translated and validated into Brazilian Portuguese (RosaQol-BR).^33^

The RosaQol-BR consists of 21 questions related to three domains, which explain how rosacea affects patients' quality of life and are related to the severity reported by patients: symptoms, emotions, and function. The result is based on an average of the responses (“never ‒ 1”, “rarely ‒ 2”, “sometimes ‒ 3”, “frequently ‒ 4” or “always ‒ 5”) related to each domain, in addition to the global assessment. The final score for each domain can therefore vary from 1 to 5, and the final value is the sum of the averages for each question.^33^ The RosaQol-BR questionnaire is self-administered.

All participants were classified according to the characteristics and severity of rosacea using the Investigator Global Assessment of Rosacea Severity Score (IGA-RSS), with the following parameters: IGA-0: Absent (rosacea almost absent ‒ absence of papules and/or pustules, absent or residual erythema, mild degree of telangiectasias may be present), IGA-1: Minimal (rare papules or pustules, residual erythema and telangiectasias may be present), IGA-2: Mild (some papules and/or pustules, mild erythema and mild or moderate degree of telangiectasias), IGA-3: Mild to moderate (distinct number of papules and/or pustules, erythema and telangiectasias of mild to moderate degrees), IGA-4: Moderate (marked number of papules and pustules, erythema and moderate degree of telangiectasias), IGA-5: Moderate to severe (many papules and/or pustules, occasionally with large inflammatory lesions, moderate erythema and moderate degree of telangiectasias), IGA-6: Severe (numerous papules and pustules, occasionally with confluent areas of inflammation, erythema and severe degree of telangiectasias).^34^

The clinical assessment of rosacea, based on the Rosacea Clinical Scorecard (RCS), includes its classification as absent, mild, moderate, or severe based on primary characteristics (flushing, persistent erythema, papules and pustules, telangiectasias), secondary characteristics (burning or stinging, plaques, dry appearance, edema, ocular manifestation, peripheral manifestation, phymatous or granulomatous changes) and the classification “absent”, “mild”, “moderate” or “severe” for the subtypes: erythematotelangiectatic, papulopustular, phymatous, ocular and global assessment.^35^

The description of the numerical variables was used for the statistical analysis, with mean and standard deviation, and, for categorical data, the absolute value and the calculation of the percentage in relation to the total sample. The internal consistency of the RosaQoL questionnaire was assessed by Cronbach's alpha coefficient, considering adequate if > 0.8.

## Results

A total of 258 patients diagnosed with rosacea were evaluated, and the main demographic data are shown in Table 1. There was a predominance of female participants (2.3 times), aged between 35 and 65 years, with phototypes II to IV (95%), with overweight and obesity (67.3%), of European descent (83%) and with a family history of rosacea (32%).

**Table 1:** Main demographic characteristics of participants with rosacea (n = 258).

Variable	Values
Age (years)^a^	51.3 ± 14.4
Body Mass Index (kg/m^2^)^a^	26.9 ± 5.7
Overweight (BMI > 25 < 30 kg/m^2^)^b^	100 (39.21%)
Obesity (BMI ≥ 30 kg/m^2^)^b^	72 (27.9%)
Time to diagnosis (year)^a^	8.0 ± 8.6
Total duration of disease (years)^a^	11.1 ± 9.2
**Sex**^b^	
Female	179 (69.4%)
Male	79 (30.6%)
**Phototype**^b^	
I	7 (2.7%)
II	61 (23.7%)
III	119 (46.3%)
IV	66 (25.7%)
V	4 (1.6%)
**Self-reported ancestry**^b^	
European	215 (83.3%)
African-American	18 (7.0%)
Eastern-Asian	2 (0.8%)
Indigenous	23 (8.9%)
**Level of schooling**^b^	
Did not attend	4 (1.6%)
Kindergarten	25 (9.7%)
Elementary School	50 (19.4%)
High School	61 (23.6%)
Higher Education	118 (45.7%)
Family history of rosacea^b^	83 (32.3%)
**Medications in use**^b^	185 (71.7%)
Anti-hypertensives	80 (31.0%)
Antidepressants	50 (19.4%)
Antidiabetics	45 (17.4%)
Lipid-lowering agents	38 (14.8%)
Levothyroxine	29 (11.3%)
Oral contraceptives	25 (9.7%)
Proton-pump inhibitor	22 (8.6%)
Antiplatelet agents	10 (3.9%)
Vasodilators	7 (2.7%)
Others^c^	87 (47%)
**Treatments used for rosacea**^b^	
Topical	167 (64.7%)
Azelaic acid	82 (31.8%)
Ivermectin	91 (35.3%)
Metronidazole	87 (33.7%)
Oral	78 (30.2%)
Cyclins	71 (27.5%)
Isotretinoin	19 (7.4%)
Laser / pulsed light	55 (21.3%)

a Mean and standard deviation.

b Absolute and percentage values.

c Medications with less than 2% frequency.

The high prevalence of use of antihypertensives (31%), antidepressants (19%), antidiabetics (17%) and lipid-lowering drugs (15%) stands out. The time of disease evolution was highly variable (from three months to 45 years, with an average of 11 years). In addition to topical interventions, 30% had already used oral treatments such as tetracyclines and isotretinoin.

According to the IGA-RSS scale, 83% of the cases had mild to moderate rosacea. The clinical score (RCS) identified flushing in 84% of cases, burning sensation in 69%, papules and pustules in 73%, phymas in 22%, and ocular forms in 41%. According to the patient's assessment, 89% classified disease activity as mild to moderate. The other clinical elements of the sample, such as rosacea severity assessment and rosacea characteristics assessed by the investigator and patients, are shown in Table 2.

**Table 2:** Clinical evaluation of rosacea using the Investigator Global Assessment of Rosacea Severity Score (IGA-RSS) and Rosacea Clinical Scorecard (RCS; n = 258).

Clinical assessment scores (investigator)	Categories	n (%)
**IGA-RSS**	1	73 (29.4%)
	2	97 (39.1%)
	3	45 (18.1%)
	4	25 (10.1%)
	5	7 (2.8%)
	6	1 (0.4%)
**RCS**		
Flushing	Absent	42 (16.3%)
	Mild	100 (38.8%)
	Moderate	93 (36.0%)
	Intense	23 (8.9%)
Erythema	Absent	67 (26%)
	Mild	122 (47.3%)
	Moderate	65 (25.2%)
	Intense	4 (1.6%)
Papules and Pustules	Absent	91 (35.3%)
	Mild	121 (46.9%)
	Moderate	43 (16.7%)
	Intense	3 (1.2%)
Telangiectasias	Absent	34 (13.2%)
	Mild	146 (56.6%)
	Moderate	71 (27.5%)
	Intense	7 (2.7%)
Burning or stinging	Absent	81 (31.4%)
	Mild	83 (32.2%)
	Moderate	73 (28.3%)
	Intense	21 (8.1%)
Plaques	Absent	219 (84.9%)
	Mild	22 (8.5%)
	Moderate	14 (5.4%)
	Intense	3 (1.2%)
Dry appearance	Absent	148 (57.4%)
	Mild	68 (26.4%)
	Moderate	40 (15.5%)
	Intense	2 (0.8%)
Edema	Absent	224 (87.2%)
	Mild	26 (10.1%)
	Moderate	5 (1.9%)
	Intense	2 (0.8%)
Ocular	Absent	151 (58.5%)
	Mild	76 (29.5%)
	Moderate	24 (9.3%)
	Intense	7 (2.7%)
Peripheral (extra-facial)	No	233 (90.7%)
	Cervical	12 (4.7%)
	Pre-auricular	1 (0.4%)
	Back	1 (0.4%)
	Scalp	3 (1.2%)
	Anterior thorax/neck	7 (2.7%)
Phymatous changes	Absent	200 (77.5%)
	Mild	23 (8.9%)
	Moderate	27 (10.5%)
	Intense	8 (3.1%)
Changes compatible with granulomatous rosacea	Absent	238 (92.2%)
	Mild	8 (3.1%)
	Moderate	11 (4.3%)
	Intense	1 (0.4%)
Erythematous telangiectatic	Absent	14 (5.4%)
	Mild	146 (56.6%)
	Moderate	89 (34.5%)
	Intense	9 (3.5%)
Papulopustular	Absent	96 (37.2%)
	Mild	119 (46.1%)
	Moderate	40 (15.5%)
	Intense	3 (1.2%)
Phymatous	Absent	197 (76.4%)
	Mild	26 (10.1%)
	Moderate	27 (10.5%)
	Intense	8 (3.1%)
Ocular	Absent	157 (60.9%)
	Mild	70 (27.1%)
	Moderate	23 (8.9%)
	Intense	8 (3.1%)
Overall patient assessment	Absent	8 (3.1%)
	Mild	129 (50.0%)
	Moderate	100 (38.8%)
	Intense	21 (8.1%)

*Results presented in absolute values and percentages.

The results of the mean scores of the domains measured by RosaQoL (internal consistency defined by Cronbach's alpha coefficient was > 0.8), self-perception of rosacea severity, and self-perception of the impact on quality of life caused by the disease are shown in Table 3. The most affected quality of life domain was symptoms. Overall, 75% of the participants considered their rosacea as good and regular over the past four weeks, and 58% considered that rosacea interfered little or reasonably with their quality of life.

**Table 3:** Quality of life in participants with rosacea, according to the RosaQoL instrument, self-perception of rosacea severity, and self-perception of quality of life inflicted by the disease (n = 258).

Variables	Values
**Domains**^a^	
Symptom	2.9 ± 0.8
Emotion	2.7 ± 1.1
Function	2.1 ± 1.0
General	2.6 ± 0.8
**How has your rosacea been over the past four weeks?**^b^	
Bad	37 (14.5%)
Regular	110 (43.3%)
Good	80 (31.3%)
Very good	18 (7.0%)
Excellent	11 (4.3%)
**How much does your rosacea influence your quality of life?**^b^	
Not at all	48 (18.7%)
A little	82 (31.9%)
Reasonably	68 (26.5%)
A lot	46 (17.9%)
Very much	13 (5.1%)

Results presented in mean and standard deviation^a^ and absolute and percentage frequencies^b^.

Aggravating factors for rosacea were reported in 96% of cases, with environmental exposure, emotional conditions, and physical exercise predominating. Food items were reported as aggravating factors by 72 (27.9%) of the participants, with pepper and other condiments and hot beverages being the most frequently mentioned (Table 4).

**Table 4:** Aggravating factors reported by participants with rosacea (n = 258).

Factors^a^	Values – n (%)
Sun exposure	196 (76.0%)
Emotional changes	169 (65.5%)
Heat	160 (62.0%)
Physical exercise	115 (44.6%)
Alcohol	110 (42.6%)
Wind	66 (25.6%)
Foods	72 (27.9%)
Pepper	39 (54.16%)^b^
Other condiments	9 (12.5%)^b^
Hot foods/beverages	6 (8.3%)^b^
Dairy products	6 (8.3%)^b^
Chocolate	6 (8.3%)^b^
Coffee	5 (6.9%)^b^
Wine	4 (5.6%)^b^
Fats/fried foods	4 (5.6%)^b^
Milk	4 (5.6%)^b^
Sugar	4 (5.6%)^b^
Sweets	3 (4.2%)^b^
Processed meats-preservatives	3 (4.2%)^b^
Crustaceans	3 (4.2%)^b^
Tomato paste/tomato	2 (2.8%)^b^
Mayonnaise	2 (2.8%)^b^
Ketchup	2 (2.8%)^b^
Ginger	2 (2.8%)^b^
Cinnamon	2 (2.8%)^b^
Others	14 (19.4%)^b^
No aggravating factors mentioned	10 (3.9%)

a There may be more than one factor for each patient.

b Percentage related to the 72 cases that reported aggravation due to food.

Certain foods traditionally recognized as aggravating factors were investigated regarding the frequency of consumption, and the following were consumed daily by the patients in the present study: hot coffee (79.1%), milk and dairy products (72.9%), tomatoes (54.3%), citrus fruits (50.4%), vegetable fat (44.5%), chocolate (19%), vinegar (11.6%), ginger (7.4%) and pepper (6.2%).

Active smoking was reported by 8% of the patients and alcoholism by 5%. Comorbidities were identified in 89.1% of the participants (Table 5), with emphasis on gastrointestinal, endocrine, cardiovascular, and psychiatric diseases.

**Table 5:** Reports of comorbidities reported by research participants (n = 258).

Comorbidities	n (%)
*Gastrointestinal condition*	71 (27.5%)
Crohn’s disease / URC	6 (2.3%)
Gastroesophageal reflux disease	32 (12.4%)
Gastritis/gastric ulcer	40 (15.5%)
Pancreatitis	1 (0.4%)
Bariatric surgery	1 (0.4%)
Lactose intolerance	5 (1.9%)
Irritable bowel syndrome	5 (1.9%)
Colitis	1 (0.4%)
Diverticulitis	1 (0.4%)
Gastric hernia	1 (0.4%)
*Neurological disease*	46 (17.8%)
Cerebrovascular accident	6 (2.3%)
Migraine	39 (15.1%)
Other neurological disease	3 (1.2%)
*Psychiatric disease*	89 (34.5%)
Depression	46 (17.9%)
Anxiety	67 (26.1%)
Social phobia	1 (0.4%)
OCD	1 (0.4%)
Panic syndrome	1 (0.4%)
Insomnia	2 (0.8%)
*Autoimmune disease*	38 (14.8%)
Psoriasis	14 (5.4%)
Lupus erythematosus	6 (2.3%)
Frontal fibrosing alopecia	4 (1.6%)
Rheumatoid Arthritis	3 (1.2%)
Vitiligo	2 (0.8%)
Scleroderma or systemic sclerosis	2 (0.8%)
Hashimoto’s disease	4 (1.5%)
Purpura and hidradenitis	1 (0.4%)
Purpura	1 (0.4%)
Pemphigus foliaceus	1 (0.4%)
Transverse myelitis	1 (0.4%)
Lupus, scleroderma and dermatomyositis	1 (0.4%)
*Infectious disease*	12 (4.7%)
HIV/AIDS	4 (1.5%)
Hepatitis B	3 (1.2%)
Chagas’ disease	1 (0.4%)
Leprosy	1 (0.4%)
Recurrent UTI	1 (0.4%)
Latent tuberculosis	1 (0.4%)
Chromomycosis	1 (0.4%)
*Neoplastic disease*	40 (15.6%)
Cutaneous BCC/SCC	23 (8.91%)
CIN 3	1 (0.4%)
Melanoma	7 (2.7%)
Larynx	1 (0.4%)
Thyroid	3 (1.16%)
Breast	5 (1.9%)
Ovarian teratoma	1 (0.4%)
Intestine / Rectum	1 (0.4%)
Prostate	2 (0.8%)
Endometrium	1 (0.4%)
Leukemia	1 (0.4%)
*Cardiovascular diseases*	81 (31.4%)
Arterial Hypertension	77 (29.8%)
Acute myocardial infarction	3 (1.2%)
Dyslipidemia	40 (15.5%)
Arrythmia / atrial fibrillation	7 (2.7%)
Cardiomegaly	1 (0.4%)
Heart failure	1 (0.4%)
Venous thrombosis	2 (0.8%)
*Endocrine disease*	123 (47.7%)
Diabetes mellitus	42 (16.3%)
Thyroid nodule	3 (1.2%)
Hypothyroidism	18 (7%)
Insulin resistance	3 (1.2%)
Osteoporosis	1 (0.4%)
Obesity	61 (23.6%)
Metabolic syndrome	42 (16.3%)
*Liver disease*	26 (10.07%)
Steatosis	22 (8.5%)
Liver thrombosis	1 (0.4%)
Post-hepatitis cirrhosis	1 (0.4%)
Drug-induced hepatitis	1 (0.4%)
Liver transplant	1 (0.4%)
*Urological disease*	21 (8.1%)
Renal lithiasis	15 (5.8%)
Glomerulonephritis ‒ transplant	1 (0.4%)
Prostatic hyperplasia	2 (0.8%)
Nephritis in childhood	1 (0.4%)
Renal cyst	1 (0.4%)
Pyelonephritis	1 (0.4%)

URC, Ulcerative rectocolitis; OCD, Obsessive-Compulsive Disorder; HIV/AIDS, Human Immunodeficiency Virus/Acquired Immunodeficiency Syndrome; UTI, Urinary Tract Infection; BCC/SCC, Basal Cell Carcinoma/Squamous Cell Carcinoma; CIN, Cervical Intraepithelial Neoplasia.

## Discussion

The multiethnic characteristics and cultural and climatic variability of Brazil require studies with representative samples from different regions for a more adequate understanding of the characteristics of its dermatoses, aiming to improve disease perception and its therapeutic approach. There are national data on the prevalence of rosacea with different results, which may reflect this variation between regions: in a study conducted in the south of the country, a higher prevalence of rosacea was found in women aged between 40 and 50 years,^32^ while in another the most affected age group was between 60 and 79 years and the least affected was under 30 years.^5^ In other non-Brazilian studies, a predominance was observed in women aged between 30 and 60 years, and it can also affect children and the elderly.^2^, ^9^, ^35^ There were also reports that men develop more severe forms, possibly due to androgenic activity.^6^, ^36^ Furthermore, a study showed a higher prevalence in female patients up to 49 years of age, but the prevalence was higher in men between 50 and 70 years of age.^37^

In this investigation, as mentioned in Table 1, a high predominance of rosacea was found among women (69.4%). An additional difference between the sexes in this study compared to others may have occurred due to the tendency for women to seek medical care more frequently.^9^, ^35^, ^38^ A wide age range affected by the disease was also identified, including cases of rosacea in individuals over 60 years of age.

There is a consensus that rosacea predominates in people with lower phototypes (I and II) according to the Fitzpatrick classification, 1988,^36^, ^39^ although there are reports of the disease in populations with higher phototypes.^35^, ^36^, ^40^ The results of the present study showed that almost half of the population studied was phototype III, followed by phototypes IV and II. The disease in phototypes I and V was rarely observed. These data demonstrate a particularity of the disease in Brazil, which may be related to the ethnic composition of the Brazilian population. It is noteworthy that the suspicion of rosacea is more difficult in darker skin, leading to a late diagnosis.

Rosacea is more frequently described in European countries, particularly in the north.^36^ The majority (83.3%) of the patients in the present study were of European descent, corroborating findings from previous Brazilian studies.^5^, ^32^, ^41^

Regarding the patients’ occupations, there was a wide diversity of professions and there was no association regarding the prevalence of rosacea in the reported professions.

As for the level of schooling, it was observed that 45.7% of the study participants had completed higher education and 23.6% had completed high school. It was not possible to compare this finding because, to date, it has not been reported in the literature on rosacea. It is important to emphasize that in Brazil there is a high presence of adult white women in higher education, which has a likely interface with this high level of schooling found in the sample.^42^

Only 8.1% of the study population were smokers. The inverse association between rosacea and smoking is controversial and some authors claim that active smoking may be a protective factor for rosacea.^5^, ^43^ There have been reports that nicotine may play an anti-inflammatory role^43^, ^44^ and promote vasoconstriction in the skin.^45^, ^46^

In the present study, 95% of the individuals were non-alcoholics; however, among them, 42.6% associated alcohol intake with rosacea exacerbation. Although it is mainly related to flushing, the association between alcoholic beverages and the onset of rosacea remains controversial in the literature.^29^, ^32^, ^47^, ^48^ A study carried out with 82,737 North American women followed for 14 years, with 4,948 cases of rosacea, showed that higher alcohol intake was associated with an increased risk of the disease compared to patients who never consumed alcoholic beverages.^47^ A recent meta-analysis concluded that alcohol consumption is not a risk factor for rosacea; however, in the subgroup analysis, a higher risk for the phymatous form was observed.^48^ Another study showed that the use of alcoholic beverages increased the risk of rosacea, but only with high consumption per week.^2^

Regarding information on height, weight, and BMI, there is little data on patients with rosacea in the literature.^44^ In the present investigation, it was found that the average weight was 73.5 kg ± 15.21; the mean height was 1.65 m ± 0.1 and the mean body mass index was 26.87 kg/m^2^ ± 5.67. Additionally, 39.21% of the participants were classified as overweight and 28.23% showed some degree of obesity. The increased risk of rosacea in patients with obesity had already been described, with a risk ratio of 1.48 in patients with BMI ≥ 35.0 when compared to those with a BMI between 21.0 and 22.9 kg/m^2^.^49^

A family history of rosacea occurred in one-third of the cases, corroborating the role of previously reported. genetic (yet to be better understood) and environmental factors^16^, ^20^, ^25^ A study with identical and fraternal twins showed a genetic participation of 46% in rosacea manifestations.^50^

Disease evolution showed an average period of 11 years, and the average time until diagnosis was eight years. Several factors can delay the diagnosis of rosacea, such as difficulty in accessing tertiary health services, overlapping symptoms with other dermatoses, and diagnosis complexity in darker skin due to difficulty in visualizing vascular manifestations, such as erythema and telangiectasia. It is noteworthy that 27% of the individuals were phototypes IV and V in the population of this study, which could be considered a factor for later diagnosis.

The use of systemic medications to treat other diseases was highly prevalent, particularly antihypertensive drugs. There are contradictory reports of worsening or triggering of rosacea by the use of calcium channel blockers, which should be avoided, and there is debate about the reduced risk in patients using beta-blockers.^51^, ^52^, ^53^

The use of antidepressants was observed in approximately 20% of individuals, and the relationship between rosacea and psychiatric disorders, especially anxiety and depression, is frequently reported.^14^, ^15^ A discrepancy was observed between rosacea severity and the impact on quality of life since the disease was moderate or severe in 46.8% of the participants and only 23% reported a negative influence on quality of life. An important consideration is the cross-sectional approach of the present study. Many patients were undergoing treatment, which positively impacted the perception of quality of life. It is known that dermatological treatment interferes with this assessment, improving the results,^54^ making it difficult to draw conclusions about the true extent of the disease impact. This dissociation between severity and quality of life has already been reported,^14^ since patients with mild forms can have a relevant impact when left untreated.

Regarding treatments, topical medications were mentioned by 64.7% of patients, and systemic medications by 30.2%. The active ingredients were in accordance with the recommendations of therapeutic guidelines for rosacea.^41^, ^55^, ^56^

Data related to the demographic characteristics of the participants in the present study can be found in Table 1.

The frequency of the observed clinical manifestations of rosacea was similar to those described in a meta-analysis,^57^ with the exception of phytates and ocular changes, which were more prevalent in the present study (Table 2).

Subjective symptoms such as burning or stinging can have a negative impact on quality of life, in addition to representing an auxiliary tool for diagnosis.^58^ The frequency of this symptom is usually under-assessed in clinical practice, due to the difficulty in measuring it. A high frequency was observed, particularly with mild or moderate intensity, as in a previous study.^58^

Chronic inflammation in rosacea has been associated with multiple comorbidities, including cardiovascular, psychiatric, neurodegenerative, gastrointestinal, neurological, oncological, endocrine, and autoimmune diseases. The extent, clinical significance, and consequences of these associations are not fully understood.^59^, ^60^ In the present sample, 27.5% of the patients reported some gastrointestinal disease; this association should be considered by physicians for early diagnosis and therapeutic implications.^61^, ^62^, ^63^

Neurovascular dysregulation and chronic inflammation may explain the association between rosacea and headache, particularly migraine.^64^ Most patients with rosacea and headache have the erythematotelangiectatic subtype and, in addition to flushing or transient erythema, persistent erythema and telangiectasias characteristic of the skin condition, photophobia and phonophobia are common. A study showed that 15% of patients with rosacea had migraines, and the risk seemed higher in men.^65^ In contrast, the present study showed a higher prevalence of migraines in women, accounting for 17.9%, while the prevalence in men was 8.9%.

Although there have been reports of an increased risk of neurodegenerative diseases in patients with rosacea, particularly Parkinson's disease and dementia, such as Alzheimer's disease,^66^ the present data did not demonstrate these conditions. The risk of developing psychiatric symptoms is 1.6 to 2-fold higher in patients with rosacea when compared to controls, possibly due to shared mechanisms involving the neuro-immuno-cutaneous system.^14^, ^15^, ^66^, ^67^ The findings of the present study are in agreement with this observation, since psychiatric diseases were detected in 34.5% of the individuals, particularly anxiety and depression.

Regarding oncological diseases, the literature shows that rosacea is associated with an increased risk of Non-Melanoma Skin Cancer (NMSC), glioma, and breast cancer.^66^, ^68^ In the present population, 23 (8.9%) patients with NMSC were detected; however, fair skin and European ancestry are common factors for rosacea and this type of skin cancer.

Patients with rosacea are predisposed to the risk of subclinical cardiovascular (CV) disease, atherosclerosis, and thromboembolism,^69^, ^70^, ^71^, ^72^, ^73^ with screening and guidance on lifestyle modifications being advised. In this study, 31.4% of patients reported CV disease, 29.8% had hypertension, and 15.5% had dyslipidemia.

Endocrinological abnormalities are frequently observed in patients with rosacea, having been found in half of the participants in this study. The main endocrine disorders were diabetes mellitus, obesity, and metabolic syndrome.

Clinicians should be aware of the potential risk of systemic comorbidities in rosacea, which becomes more likely as the duration and severity of the disease increase. The potential to control these conditions aiming to improve rosacea symptoms and vice versa should be the focus of further research.^13^, ^66^

Quality of life is impacted in most patients with rosacea, since the disease is chronic, recurrent, and manifests itself in exposed areas, such as the face. Low self-esteem, social phobia, depression, and anxiety may occur, which compromise the affected individuals’ mental health and socialization.^74^, ^75^ The RosaQoL-BR scores revealed an impact on the quality of life in almost 50% of patients, although almost 90% considered their disease to be mild to moderate in the overall assessment, as it was considered by the researchers.

Several environmental factors are described as triggering or aggravating rosacea, which were reported by 96% of the participants in this study. As shown in Table 4, which lists the triggering or aggravating factors reported by the study participants, sun exposure was the main one, corroborating the data in the literature.^76^, ^77^ The higher disease prevalence in lighter skin and the presence of lesions in the central area of the face, which are the most exposed to sunlight, corroborate this hypothesis.^3^ UVR can trigger rosacea, actively participating in its etiopathogenesis.^77^

Acute emotional changes were reported by more than half of the participants, reinforcing stress as a relevant trigger, as it increases levels of cortisol and adrenocorticotropic hormone, activating the hypothalamic-pituitary-adrenal axis.^77^

Exposure to heat was mentioned as a trigger by 62% of the participants, as well as, to a lesser extent, cold and wind. It is important to highlight the great climatic variation in the different regions of Brazil, such as extreme heat in the Amazon region, cold in the subtropical regions, and heat and wind on the coast of the Northeast. This study included patients from states in all of these regions, making the sample quite heterogeneous, but representative of the country's climate variability. Disease worsening due to physical exercise, which has a thermogenic and vasodilatory effect, was reported by almost half of the participants.^3^, ^76^

The intake of certain foods is referred to as a trigger or worsening factor for rosacea, although there are few studies confirming this property.^18^, ^19^ In this study, 28% of the participants reported worsening of rosacea with the diet, with pepper being reported by more than half of them, followed by other spicy foods and seasonings. The foods considered as triggers can be divided into four categories: hot foods/beverages, such as coffee (33%) and tea (30%), spicy foods, foods with cinnamaldehyde, and alcoholic beverages.^18^, ^76^ Capsaicin is present in peppers and cinnamaldehyde is found in tomatoes, citrus fruits, cinnamon, and chocolate. The relationship between diet and the gut-skin axis has been reported, interfering in the clinical manifestations of the disease.^18^

The present results demonstrated that some foods that are consumed daily were rarely mentioned as triggers of the disease, such as hot coffee, milk and dairy products, tomatoes, and citrus fruits. Contrary to what was believed in the past, a cohort study with 82,737 women suggests that caffeine may have a protective effect on rosacea, reducing symptoms by inducing a vasoconstrictor and immunosuppressive response.^79^ The influence of milk and dairy products is controversial, with a possible negative correlation, suggesting that they are protective in the erythematotelangiectatic and papulopustular phenotypes.^19^ Daily consumption of tomatoes and citrus fruits was reported by more than half of the studied population, although they were not mentioned as aggravating factors. To date, there is no evidence that eliminating certain foods results in an improvement in rosacea symptoms.^78^ However, for Brazilians, this study suggests that the consumption of alcoholic beverages and diets containing pepper may be important in worsening the disease, based on the frequency of ingestion and the patient’s perception.

This study has limitations, especially related to the lack of a control group, the inclusion of patients from referral centers, and the fact that many were undergoing treatment. Other observational studies and, mainly, population-based studies are necessary to confirm the findings presented herein.

## Conclusion

The higher prevalence of rosacea in phototypes III and IV in the Brazilian sample studied demonstrates the need for dermatologists to be alert to signs of the disease, since diagnosis may be delayed in higher phototypes.

The assessment of comorbidities plays a fundamental role, particularly regarding overweight and obesity. Cardiovascular, psychiatric, neurodegenerative, gastrointestinal, neurological, oncological, endocrinological, and autoimmune diseases were detected, demonstrating that the identification of these comorbidities can allow screening and adequate monitoring of patients.

Gastrointestinal manifestations were highly prevalent, corroborating the possible relationship between rosacea, microbiome and gastrointestinal tract diseases (gut-skin axis).

The quality of life impairment and high prevalence of psychiatric diseases demonstrate the importance of addressing these aspects.

In the present sample, certain foods and the use of alcoholic beverages were mentioned as aggravating factors of the disease, and specific research is required to determine the impact of diet on rosacea.

This study presents real data from different regions of Brazil, which may contribute to the development of algorithms for the holistic management of patients with rosacea.

## Financial support

FIPE (Fundo de Incentivo à Pesquisa e Eventos ‒ Hospital de Clínicas de Porto Alegre, Brazil).

## Authors' contributions

Renan Rangel Bonamigo: Design and planning of the study; collection and analysis of data; drafting and editing of the manuscript; review of the literature; approval of the final version of the manuscript.

Paula Barea: Collection and analysis of data; assembly of the database; drafting and editing of the manuscript; review of the literature; approval of the final version of the manuscript.

Juliano Peruzzo: Design and planning of the study; collection and analysis of data; drafting and editing of the manuscript; review of the literature; approval of the final version of the manuscript.

Juliana Boza: Design and planning of the study; collection and analysis of data; drafting and editing of the manuscript; review of the literature; approval of the final version of the manuscript.

Hélio Amante Miot: Design and planning of the study; collection of data; drafting and editing of the manuscript; review of the literature; approval of the final version of the manuscript.

Edileia Bagatin: Design and planning of the study; collection of data; drafting and editing of the manuscript; review of the literature; approval of the final version of the manuscript.

Luiz Maurício Costa Almeida: Design and planning of the study; collection of data; drafting and editing of the manuscript; review of the literature; approval of the final version of the manuscript.

Giovanni Indelicato Milano: Collection of data; drafting and editing of the manuscript.

Carla Wanderley Gayoso de Lima: Design and planning of the study; collection of data; drafting and editing of the manuscript; review of the literature; approval of the final version of the manuscript.

Linácia Freitas Vidal: Collection of data; drafting and editing of the manuscript.

Fabiola Rosa Picosse: Design and planning of the study; collection of data; drafting and editing of the manuscript; review of the literature; approval of the final version of the manuscript.

Bruna Manuella de Figueiredo Afonso: Collection of data; drafting and editing of the manuscript.

Clivia Maria Moraes de Oliveira: Design and planning of the study; collection of data; drafting and editing of the manuscript; review of the literature; approval of the final version of the manuscript.

## Conflicts of interest

Renan Rangel Bonamigo: None declared.

Paula Baréa: None declared.

Juliano Peruzzo: None declared.

Juliana Boza: None declared.

Hélio Miot: Advisory Board – Johnson & Johnson, L’Oréal, Theraskin, Sanofi and Pfizer; Clinical Research – Abbvie, Pierre Fabre; Galderma and Merz.

Edileia Bagatin: Farmoquímica/Melora, Beiersdorf/Eucerin, L'Oreal (Vichy/La Roche Posay, Pierre-Fabre (Avene, Ducray), Theraskin, Allergan/Abbvie, USK, LeoPharma, Ache, Pechoin.

Luiz Mauricio Costa Almeida: Advisory Board – Galderma, Beiersdorf, L'Oreal, Leo Pharma, FQM Melora, Pierre Fabre and Bayer.

Giovanni Indelicato Milano: None declared.

Carla Wanderley Gayoso de Lima: None declared.

Linácia Freitas: None declared.

Fabíola Rosa Picosse: None declared.

Bruna Manuella de Figueiredo Afonso: None declared.

Clivia Maria Moraes de Oliveira: Janssen, Novartis, Sanofi, Abbvie, Galderma, L'Oreal, Boehringer.
